# A Pilot Study Examining the Dielectric Response of Human Forearm Tissues

**DOI:** 10.3390/bios13110961

**Published:** 2023-10-29

**Authors:** Yang Yu, Anubha Manju Kalra, Gautam Anand, Andrew Lowe

**Affiliations:** Institute of Biomedical Technologies, Auckland University of Technology, Auckland 1010, New Zealand; yyu@aut.ac.nz (Y.Y.); gautam.anand@aut.ac.nz (G.A.); andrew.lowe@aut.ac.nz (A.L.)

**Keywords:** multi-frequency bioimpedance analysis (MF-BIA), forearm, Cole model, dielectric properties, finite element method

## Abstract

This work aims to describe the dielectric behaviors of four main tissues in the human forearm using mathematical modelling, including fat, muscle, blood and bone. Multi-frequency bioimpedance analysis (MF-BIA) was initially performed using the finite element method (FEM) with a 3D forearm model to estimate impedance spectra from 10 kHz to 1 MHz, followed by a pilot study involving two healthy subjects to characterize the response of actual forearm tissues from 1 kHz to 349 kHz. Both the simulation and experimental results were fitted to a single-dispersion Cole model (SDCM) and a multi-dispersion Cole model (MDCM) to determine the Cole parameters for each tissue. Cole-type responses of both simulated and actual human forearms were observed. A paired *t*-test based on the root mean squared error (RMSE) values indicated that both Cole models performed comparably in fitting both simulated and measured bioimpedance data. However, MDCM exhibited higher accuracy, with a correlation coefficient (R^2^) of 0.99 and 0.89, RMSE of 0.22 Ω and 0.56 Ω, mean difference (mean ± standard deviation) of 0.00 ± 0.23 Ω and −0.28 ± 0.23 Ω, and mean absolute error (MAE) of 0.0007 Ω and 0.2789 Ω for the real part and imaginary part of impedance, respectively. Determining the electrical response of multi-tissues can be helpful in developing physiological monitoring of an organ or a section of the human body through MF-BIA and hemodynamic monitoring by filtering out the impedance contributions from the surrounding tissues to blood-flow-induced impedance variations.

## 1. Introduction

Bioelectrical impedance analysis (BIA) is a non-invasive technique that originated in the early 1930s and 1940s [1,2], involving the measurement of the electrical impedance of a tissue region [3]. Bioimpedance measurement (BIM) provides information about the physical and electrochemical processes in the tissue region and hence can be used for monitoring physiological properties and variations [4]. BIA has been widely utilized for body composition [5,6,7,8] and developed to track pulse wave propagation [9,10,11,12] and estimate the arterial diameter change [13,14,15,16]. The fundamental principle of BIM is that a small amount of alternating current is applied through the outer pair of electrodes (current-carrying electrodes), and the voltage in response is measured through the same or a different inner pair of electrodes (pick-up electrodes). The ratio of the output voltage to the input current yields bioimpedance, reflecting the dielectric response of tissues during the measurement. BIA is carried out as either single-frequency BIA (SF-BIA) or multi-frequency BIA (MF-BIA). SF-BIA finds applications in impedance cardiography (ICG) [17] and impedance plethysmography (IPG) [18]. MF-BIA is used in electrical impedance tomography (EIT) for medical imaging diagnostics [19,20,21] and bioimpedance spectroscopy (BIS), which can be used to identify the complex dielectric behaviors of human tissues [22].

Schwan [23] initially presented the frequency-dependent electrical parameters of tissues in terms of conductivity and permittivity. The electrical behaviors of human tissues can be categorized into three dispersion regions: α-dispersion (10 Hz–10 kHz), β-dispersion (10 kHz–100 MHz) and γ-dispersion (100 MHz–100 GHz) regions. Human tissues exhibit complex dielectric behavior in β-dispersion region, which is generally described through complex conductivity and permittivity. Cole and Cole [24,25] proposed an empirical model to represent the complex dielectric behavior through distributed relaxation phenomenon, resulting in the Cole–Cole relation:

(1) $$\varepsilon \left(\omega \right)=\varepsilon_{\infty }+\frac{\varepsilon_{0}-\varepsilon_{\infty }}{1+{\left(j\omega \tau \right)}^{\alpha }}$$

where ε represents the complex dielectric constant, $\varepsilon_{\infty }$ is the dielectric constant at infinite frequency, $\varepsilon_{0}$ is the dielectric constant at zero frequency, ω is the angular frequency and τ is a time constant. α is the coefficient of relaxation, which has a value between 0 and 1, depending on the nature of the material. The parameter becomes smaller as the width of the dispersion increases.

Another way of representing the Cole model is in the form of impedance expressed in terms of complex permittivity and complex conductance:

(2) $$Z\left(\omega \right)=R_{\infty }+\frac{R_{0}-R_{\infty }}{1+{\left(j\omega \tau \right)}^{\alpha }}$$

where $R_{0}$ and $R_{\infty }$ are the resistance at zero and infinite frequency, respectively. $Z\left(\omega \right)$ has a non-linear relationship with frequency, which generates a suppressed semi-circle in the impedance plane. Equation (2) has been extensively used for single-dispersion Cole modelling in MF-BIA. The Cole impedance model has been applied to BIA before and after biceps exercise [26], uncovering noteworthy variations in impedance parameters that provide insights into fatigue-induced tissue transformations. In another investigation, the four-electrode technique was employed to assess electrical impedance within hepatic tumors and the adjacent liver tissue. The outcomes reveal substantial disparities in conductivity between tumor and healthy tissue, presenting valuable implications for bioelectric applications in tissue diagnosis and therapeutic interventions [27].

The application of MF-BIA for hemodynamic monitoring has been investigated by several previous studies using computational simulation [28], tissue phantom experiments [13,29] and human subject measurement [15]. The objective of this study is to present a mathematical modelling approach where the dielectric properties of forearm tissues will be identified through MF-BIA. In this study, the finite element method (FEM) was carried out to simulate a 3D human forearm model with four tissue domains, including fat, muscle and blood-filled artery (radial artery) and bones (radius and ulna), aiming to evaluate the dielectric response and the electric field (E-field) distribution within individual tissues of the forearm. Moreover, pilot experimentation was implemented with two healthy human participants using MF-BIA at the forearm. Both simulated and measured impedance values were fitted to the single-dispersion Cole model (SDCM) and the multi-dispersion Cole model (MDCM) to elucidate tissue responses and dielectric relaxation in individual tissues. The SDCM simplifies bioimpedance modeling, assuming a single relaxation time for all tissues, while the MDCM offers more comprehensive representation by considering multiple relaxation processes within tissues.

## 2. Methodology

### 2.1. Computational Simulation

#### 2.1.1. 3D Human Forearm

**Figure 1:**
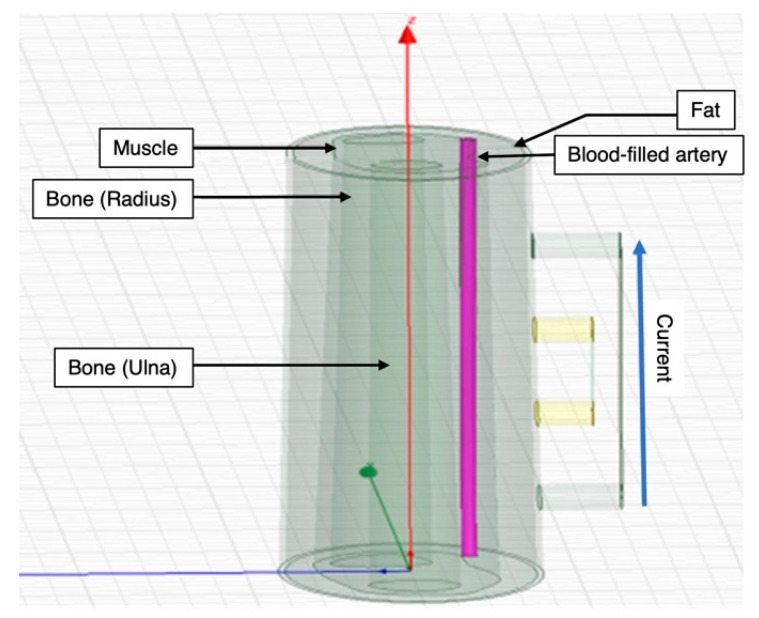
Simulated 3D human forearm in ANSYS HFSS (longitudinal view).

A 3D model of the human forearm was designed in the HFSS design type in ANSYS Electronics Desktop (2021 R2, ANSYS, Inc., Canonsburg, PA, USA), and FEM was applied to perform electrical simulation, as shown in Figure 1. In this simulation, the model encompassed five distinct tissue domains, mirroring the anatomical structure of the human forearm [30,31]: skin, fat, muscle, blood and bone. Skin contributes significantly to overall impedance measurements due to its high impedance. However, the use of wet electrodes, a common practice in BIA, was expected to mitigate the impedance effects of the outermost skin layer. Consequently, the thin outer skin layer was assigned electrical properties equivalent to those of fat tissue.

The overall width of the cross-section of the human forearm model was 70 mm. The fat and the muscle layers were modelled as concentric along the same axis. The dimension in terms of thickness of the fat was from 3 to 6 mm and from 10 to 15 mm from muscle. Additionally, a section was designated to emulate the radial artery, with a diameter of 2.35 mm, passing through both the fat and muscle domains [32,33,34,35]. The artery wall, assigned muscle properties, formed the interface between muscle and blood. The bone domain was subdivided into two main regions, namely radius, which was at the center and thicker, and ulna, which was relatively at the periphery and smaller in terms of cross-section.

Each tissue domain was assigned frequency-dependent material properties in the form of bulk conductivity (σ) and relative permittivity ($\varepsilon_{r}$), based on the database developed by Gabriel et al. [36,37,38,39]. 

#### 2.1.2. BIM Setup

Four cylindrical electrodes were modelled to simulate the tetrapolar BIM. The outer pair of electrodes was used as a current source, while the inner pair of electrodes were used for measuring the resultant potential difference. A current excitation was applied to current-carrying electrodes between 1 kHz and 1 MHz. The amplitude of the current was 1 mA in accordance with the electrical safety limits identified by IEC 60,601 standards [40]. This investigation focused on a significant portion of the β-dispersion frequency range relevant to human tissue dielectric properties, including resistance and reactance. The electrodes were chosen to be conductive by defining their material properties as those of copper. All electrodes were designed to replicate the dimensions of dry Ag/AgCl electrodes, featuring an 8 mm diameter with equidistant spacing of 20 mm. 

To obtain the simulated impedance data, a non-model line was drawn between the centers of the two pick-up (PU) electrodes. The real and imaginary parts of the complex electric field (E-field) were separately exported at 0.1 mm intervals at each frequency. Next, the real and imaginary parts of the potential difference were consequently calculated by computing the approximate cumulative integral of the real part of the E-field ($\vec{Re(E)}$) and imaginary part of the E-field ($\vec{Im(E)}$) via the trapezoidal method using MATLAB (R2019a, The MathWorks, Natick, MA, USA).

(3) $$Re(U)=\int \vec{Re(E)}\cdot d\vec{l}$$

(4) $$Im(U)=\int \vec{Im(E)}\cdot d\vec{l}$$

where $d\vec{l}$ was the vector between two adjacent points (0.1 mm). Then, the real and imaginary part of impedance “measured” by PU electrodes can be calculated by dividing the potential difference by the amount of excitation current (I = 1 mA).

### 2.2. Pilot Experimentation 

**Figure 2:**
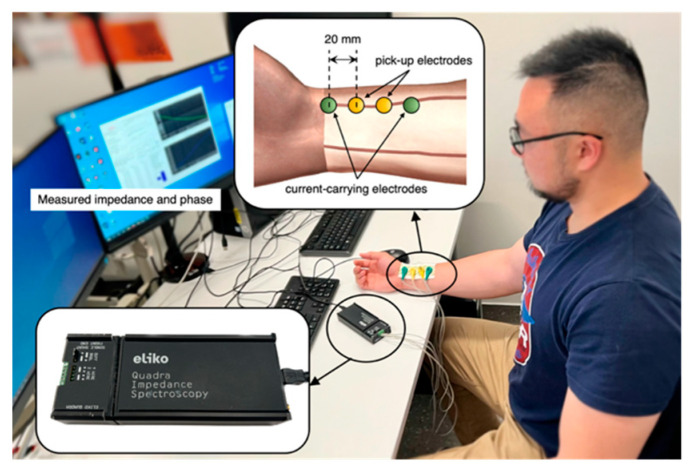
Experimental setup for BIA.

The tetrapolar BIM was implemented on two healthy subjects (as listed in Table 1) under the resting condition who were members of this research team. After consultation with the institutional ethics committee, the scope of this investigation was deemed exempt from ethics approval. As shown in Figure 2, four ECG gel electrodes (FIAB, Florence, Italy) were placed along the radial artery near the wrist of the ventral forearm. The electrode spacing was maintained at 20 mm, consistent with the computational simulation. A commercially available Quadra^®^ Electrical Impedance Spectroscopy device (Eliko^®^ technologies, Tallinn, Estonia) [41] was utilized for BIA at the Institute of Biomedical Technologies (IBTec), Auckland University of Technology, New Zealand. The Quadra^®^ device is known to have reasonable accuracy, with a system accuracy of up to 0.1%, although this accuracy may vary depending on the specific measurement and frequency range.

The reliability of the measurements was a crucial concern. Therefore, for each subject, measurements were taken over a 10 s period to ensure the consistency of the obtained spectra. This duration resulted in 10,000 samples of spectra for each frequency. From these samples, an average spectrum was calculated for each subject, demonstrating the measurement consistency in relation to changing conditions over time.

The Quadra^®^ device provides a state-of-the-art platform for impedance spectroscopy, offering a sampling rate of 1000 Hz, while yielding impedance spectra at 15 different frequencies from 1 kHz to 349 kHz. In this study, we employed extrapolation techniques to extend the observed impedance response beyond the maximum frequency limit of the Quadra^®^ device, which operates up to 349 kHz. The rationale behind this approach is rooted in the desire to obtain a more comprehensive understanding of impedance behaviors across a broader frequency spectrum. Extrapolation in MF-BIA is a well-established technique commonly employed to extend data beyond the measured range, estimate values between measured data points, or fill in gaps within a specified range [42,43,44]. It allows for a more thorough exploration of impedance characteristics, particularly in scenarios where the measurement equipment has inherent frequency limitations.

A single shunt front-end with a 4-wire differential mode was employed with the Quadra^®^ device for this study. The Quadra^®^ device interfaces V1.4 via its software, which logs the impedance in terms of absolute value (|Z|) and phase angle (θ). Therefore, the real and imaginary parts of measured impedance were calculated as follows:

(5) $$Re\left(Z\right)=\left|Z\right|\cos\left(\frac{\pi \theta }{180}\right)$$

(6) $$Im\left(Z\right)=\left|Z\right|\sin\left(\frac{\pi \theta }{180}\right)$$

### 2.3. Single and Multi-Dispersion Cole Lumped-Parameter Models

Equation (2) defines the impedance magnitude as a complex quantity and can be rewritten by substituting the value of ${\left(j\right)}^{\alpha }=\cos\left(\alpha \frac{\pi }{2}\right)+j\sin\left(\alpha \frac{\pi }{2}\right)$:

(7) $$Z\left(\omega \right)=R_{\infty }+\frac{R_{0}-R_{\infty }}{1+{\left(\omega \tau \right)}^{\alpha }cos\left[\alpha \frac{\pi }{2}\right]+i({\omega \tau )}^{\alpha }sin\left[\alpha \frac{\pi }{2}\right]}$$

The real ($Re\left(Z\right)$) and the imaginary parts ($Im\left(Z\right)$) of impedance can be obtained as follows:

(8) $$Re\left(Z\right)=R_{\infty }+\frac{\left(R_{0}-R_{\infty }\right)\left(1+{\left(\omega \tau \right)}^{\alpha }cos\left[\alpha \frac{\pi }{2}\right]\right)}{1+2{\left(\omega \tau \right)}^{\alpha }cos\left[\alpha \frac{\pi }{2}\right]+{\left(\omega \tau \right)}^{2\alpha }}$$

(9) $$Im\left(Z\right)=-j\frac{\left(R_{0}-R_{\infty }\right){\left(\omega \tau \right)}^{\alpha }sin\left[\alpha \frac{\pi }{2}\right]}{1+2{\left(\omega \tau \right)}^{\alpha }cos\left[\alpha \frac{\pi }{2}\right]+{\left(\omega \tau \right)}^{2\alpha }}$$

Equations (8) and (9) can be further combined to relate Im(Z) directly in terms of Re(Z) so as to form an equation where Im (Z) is a function of Re(Z). To aid the understanding, Re (Z) can be symbolized as ‘R’ and Im (Z) can be symbolized as ‘X’:

(10) $$\begin{array}{l} \;\;4X+2cot\left[\frac{\alpha \pi }{2}\right]\left(R_{0}-R_{\infty }\right)=\sqrt{2}cosec\left[\frac{\alpha \pi }{2}\right]*\\ \sqrt{-4R^{2}+4RR_{0}+R_{0}^{2}+4RR_{\infty }-6R_{0}R_{\infty }+R_{\infty }^{2}+cos\left[\alpha \pi \right]{\left(-2R+R_{0}+R_{\infty }\right)}^{2}} \end{array}$$

The methodology for curve fitting involved selecting a general dielectric Cole model with at least four parameters ($R_{0}$, $R_{\infty },$ τ and α) to define the dielectric relaxation spread within the frequency range of interest (i.e., from 1 kHz to 1 MHz). Furthermore, a minimization cost function was implemented, based on the sum of squared errors between the curve fit and simulation data, expressed as follows:

(11) $$RMSE=\sqrt{\frac{\sum_{i=1}^{N}{\left(R_{fit}^{i}-R_{sim}^{i}\right)}^{2}}{N}}+\sqrt{\frac{\sum_{i=1}^{N}{\left(X_{fit}^{i}-X_{sim}^{i}\right)}^{2}}{N}}$$

where RMSE is the root mean squared error; $R_{fit}^{i}$ and $X_{fit}^{i}$ refer to the R and X curve fit data for ith sample, respectively; $R_{sim}^{i}$ and $X_{sim}^{i}$ refer to the R and X simulation data for ith sample; and N is the number of samples (fitted data). The fitting algorithms were implemented using MATLAB. 

Bone exhibited an almost constant conductivity of 0.02 S/m across the entire frequency range of interest. Consequently, the contribution of the bone tissue domains was treated as resistive. In contrast, each of the other three tissue domains was characterized using a multi-dispersion Cole model, which serves as an advanced bioimpedance modeling framework capable of accommodating the complex electrical responses exhibited by biological tissues. Essentially, the multi-dispersion Cole model characterizes tissue impedance as a combination of multiple relaxation times, providing it with the capacity to replicate a wide spectrum of electrical behaviors. The final model chosen to represent the electrical response of the forearm tissues was formulated as follows:

(12) $$Z\left(\omega \right)=R_{bone}+\frac{R_{0f}-R_{\infty f}}{1+{\left(i\omega \tau_{f}\right)}^{\alpha_{f}}}+\frac{R_{0m}-R_{\infty m}}{1+{\left(i\omega \tau_{m}\right)}^{\alpha_{m}}}+\frac{R_{0b}-R_{\infty b}}{1+{\left(i\omega \tau_{b}\right)}^{\alpha_{b}}}$$

In Equation (12), the first term signifies the resistance of the bone tissues (representing both the radius and ulna), while the second, third and fourth terms elucidate the dispersion characteristics of the fat, muscle and blood tissue layers, respectively.

The evaluation of the correlation between simulated/measured impedance and the fitted impedance involved several key metrics, including the determination coefficient (R^2^) and the root mean square error (RMSE). To provide further insight into the accuracy of the fitting process, we computed both the mean difference and standard deviation (SD), which were subsequently visualized using Bland–Altman analysis. Furthermore, the mean absolute error (MAE) was employed as an additional metric to assess fitting precision. In order to determine any significant disparities in the goodness-of-fit measures, we conducted a paired *t*-test, with a specific focus on RMSE, to compare the performance of the SDCM and MDCM.

## 3. Results

### 3.1. Computational Simulation Analysis

#### 3.1.1. E-Field Distribution

**Figure 3:**
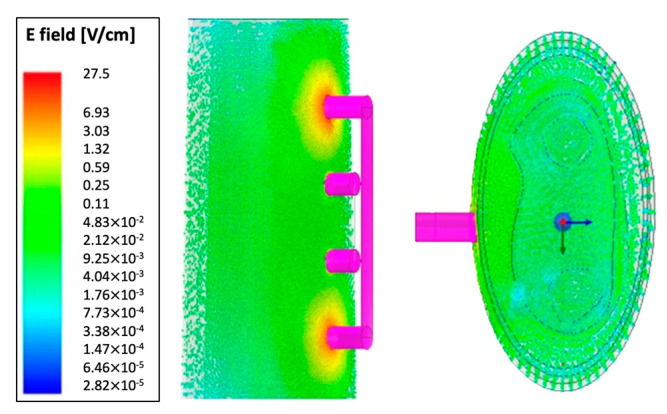
Electric field distribution throughout the 3D human forearm at 1 kHz.

Figure 3 provides a visual representation of the simulated E-field distribution across the entire 3D forearm model at a frequency of 1 kHz. This distribution of the E-field plays a pivotal role in defining the path of current flow within the model. Notably, the maximum E-field intensity is concentrated in the vicinity of the current-carrying electrodes, positioned at the outer boundaries of the model. As one moves away from these electrodes, the E-field gradually diminishes in magnitude within the surrounding regions.

#### 3.1.2. Fitting Results

**Figure 4:**
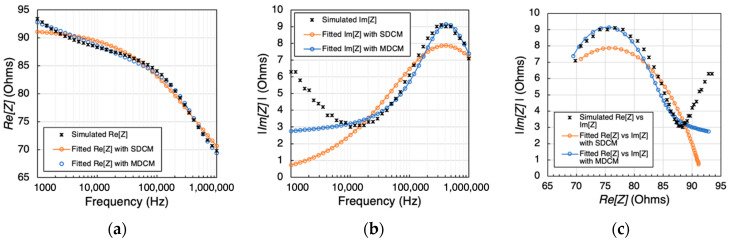
Comparison between the simulated raw data and fitted data using SDCM and MDCM: (**a**) real part of impedance; (**b**) imaginary part of impedance; (**c**) Nyquist plot (Cole plot).

Both the SDCM and the MDCM were employed to characterize the overall dielectric dispersion exhibited by the combined tissue domains. Figure 4 presents a comparative view of the simulated impedance values before and after fitting. It is noteworthy that the *Re*(*Z*), displayed a decreasing trend with increasing frequency, corresponding to the heightened conductivity of human tissues at higher frequencies. The Nyquist plot, often referred to as the Cole plot, provided insights into the Cole-type dielectric response, as evidenced by the variations in *Re*(*Z*) and *Im*(*Z*) with frequency. Particularly, the major portion of the plots, spanning from 10 kHz to 1 MHz, exhibited a semi-circular shape consistent with the Cole model, signifying the bio-tissue behavior of the simulated 3D model. However, a deviation from the Cole model became apparent at frequencies below 10 kHz. The estimated Cole parameters are shown in Table 2 and Table 3, respectively.

Then, the models describing the overall dielectric behavior can be written as follows:

(13) $$Z\left(\omega \right)=60.02+\frac{29.48}{1+{\left(j*\omega *{3.45*10}^{-7}\right)}^{0.6923}}$$

(14) $$Z\left(\omega \right)=49.72+\frac{17.29}{1+{\left(i\omega *1.48\times 10^{-9}\right)}^{0.34}}+\frac{20.46}{1+{\left(i\omega *4.08\times 10^{-7}\right)}^{0.83}}+\frac{3.5}{1+{\left(i\omega *1.5\times 10^{-4}\right)}^{0.26}}$$

**Figure 5:**
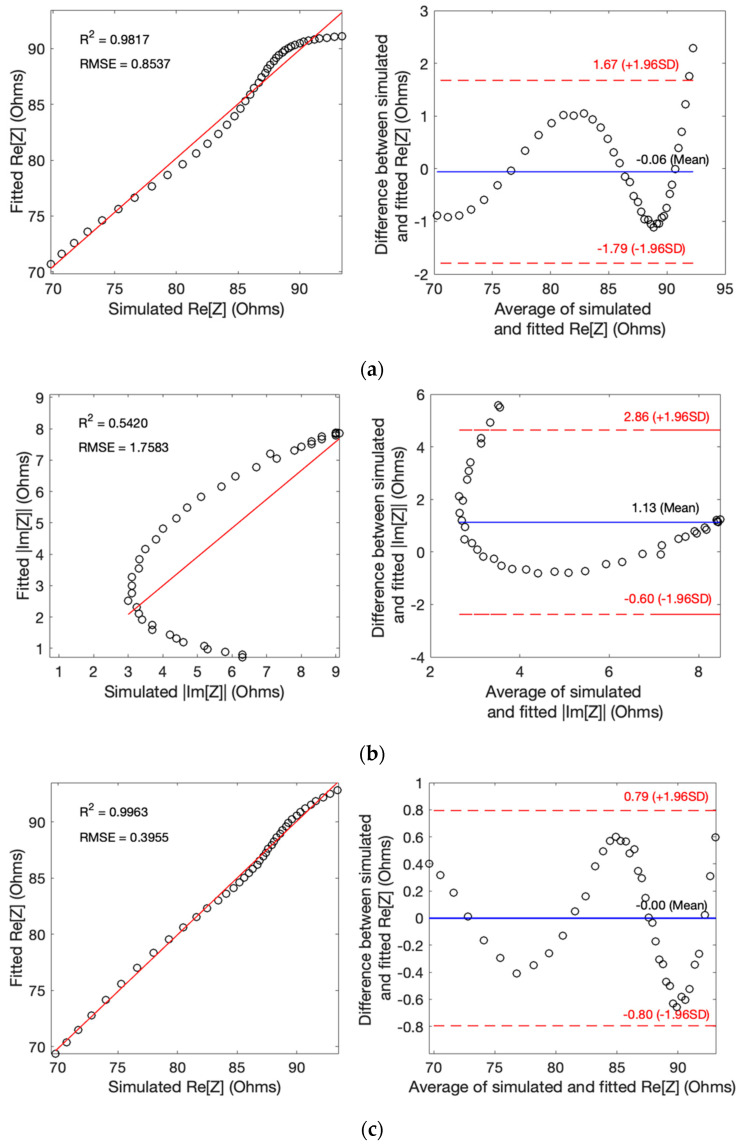

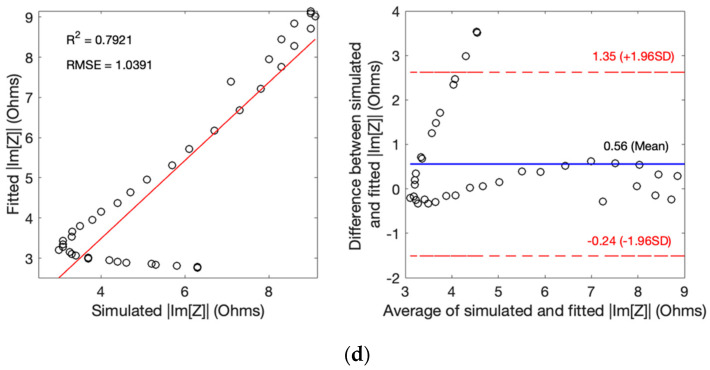
Correlation and Bland–Altman plots for simulated impedance: (**a**) real part of impedance using SDCM; (**b**) imaginary part of impedance using SDCM; (**c**) real part of impedance using MDCM; (**d**) imaginary part of impedance using MDCM.

Figure 5a,b presents correlation plots (left) and Bland–Altman plots (right) comparing raw simulated impedance with fitted impedance using the SDCM. For SDCM, Pearson’s correlation coefficients were 0.98 and 0.52, with RMSE values of 0.85 Ω and 1.75 Ω for Re[Z] and Im[Z], respectively. Bland–Altman analysis revealed mean differences of −0.06 ± 0.88 Ω and 1.13 ± 0.88 Ω Re[Z] and Im[Z], respectively, within ±1.96 SD limits representing a 95% confidence interval. Additionally, MAE values were calculated, yielding 0.0588 Ω and 1.1303 Ω for Re[Z] and Im[Z], respectively.

According to Figure 5c,d, MDCM demonstrated higher *R^2^* of 0.99 and 0.79 and lower RMSE values of 0.39 Ω and 1.04 Ω Re[Z] and Im[Z], respectively. Bland–Altman analysis indicated smaller mean differences between raw and fitted data, amounting to 0.00 ± 0.40 Ω and 0.56 ± 0.40 Ω Re[Z] and Im[Z], respectively. Furthermore, MDCM exhibited reduced MAE values of 0.0006 Ω and 0.5570 Ω Re[Z] and Im[Z], respectively. These results underscore the superior performance of the MDCM approach in accurately modeling the impedance data.

### 3.2. Pilot Study

#### Measured Impedance

**Figure 6:**
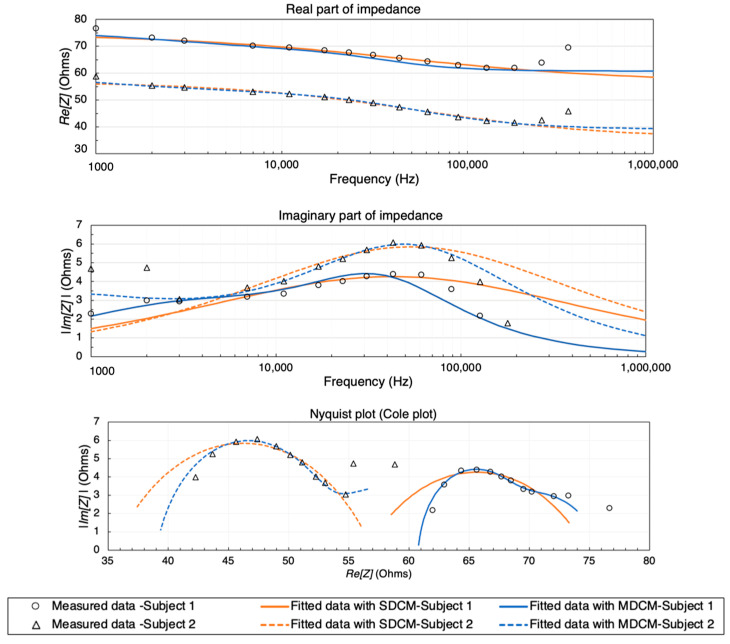
Comparison between the measured raw data and complete response from 1 kHz to 1 MHz fitted data using SDCM and MDCM for two subjects.

As shown in Figure 6, the two subjects exhibited similar impedance changes with frequency, while the impedance ranges were different due to different forearm sizes and proportions of tissues between individuals (see Table 1).

First, the measured real part and imaginary part of impedance from two subjects were fitted using both SDCM and MDCM. In contrast to the simulation setup, the measured impedance spectra were extrapolated and estimated across the entire frequency range of interest (1 kHz to 1 MHz) due to the restricted measurement capabilities of the Quadra^®^ device. Figure 6 illustrates the complete dielectric responses of forearm tissues, spanning from 1 kHz to 1 MHz, for two subjects. These responses are depicted with both solid and dashed lines, corresponding to the fitting and estimation results obtained using two different Cole models. In order to model the experimental observations of both subjects in the MDCM, it was assumed that the effect of skin (skin–electrode polarization) was countered by using gel electrodes. For the remainder of the tissues, Equation (12) was used to represent other main tissue domains. The Cole parameters of each subject were thereby estimated and tabulated in Table 4 and Table 5, respectively.

Additionally, the overall Cole models for the two subjects were thereby demonstrated:

(15) $$Z_{subject1}\left(\omega \right)=56.32+\frac{18.97}{1+{\left(j\omega \times {4.004\times 10}^{-6}\right)}^{0.54}}$$

(16) $$Z_{subject2}\left(\omega \right)=35.18+\frac{21.41}{1+{\left(j\omega \times {2.73\times 10}^{-6}\right)}^{0.64}}$$

(17) $$Z_{subject1}\left(\omega \right)=57.98+\frac{9.42}{1+{\left(i\omega \times 7.29\times 10^{-5}\right)}^{0.65}}+\frac{8.68}{1+{\left(i\omega \times 6.91\times 10^{-5}\right)}^{0.67}}+\frac{2.92}{1+{\left(i\omega \times 1.63\times 10^{-4}\right)}^{0.62}}$$

(18) $$Z_{subject2}\left(\omega \right)=38.83+\frac{13.29}{1+{\left(i\omega \times 2.32\times 10^{-4}\right)}^{0.72}}+\frac{12.21}{1+{\left(i\omega \times 3.91\times 10^{-5}\right)}^{0.88}}+\frac{2.33}{1+{\left(i\omega \times 6.28\times 10^{-4}\right)}^{0.66}}$$

**Figure 7:**
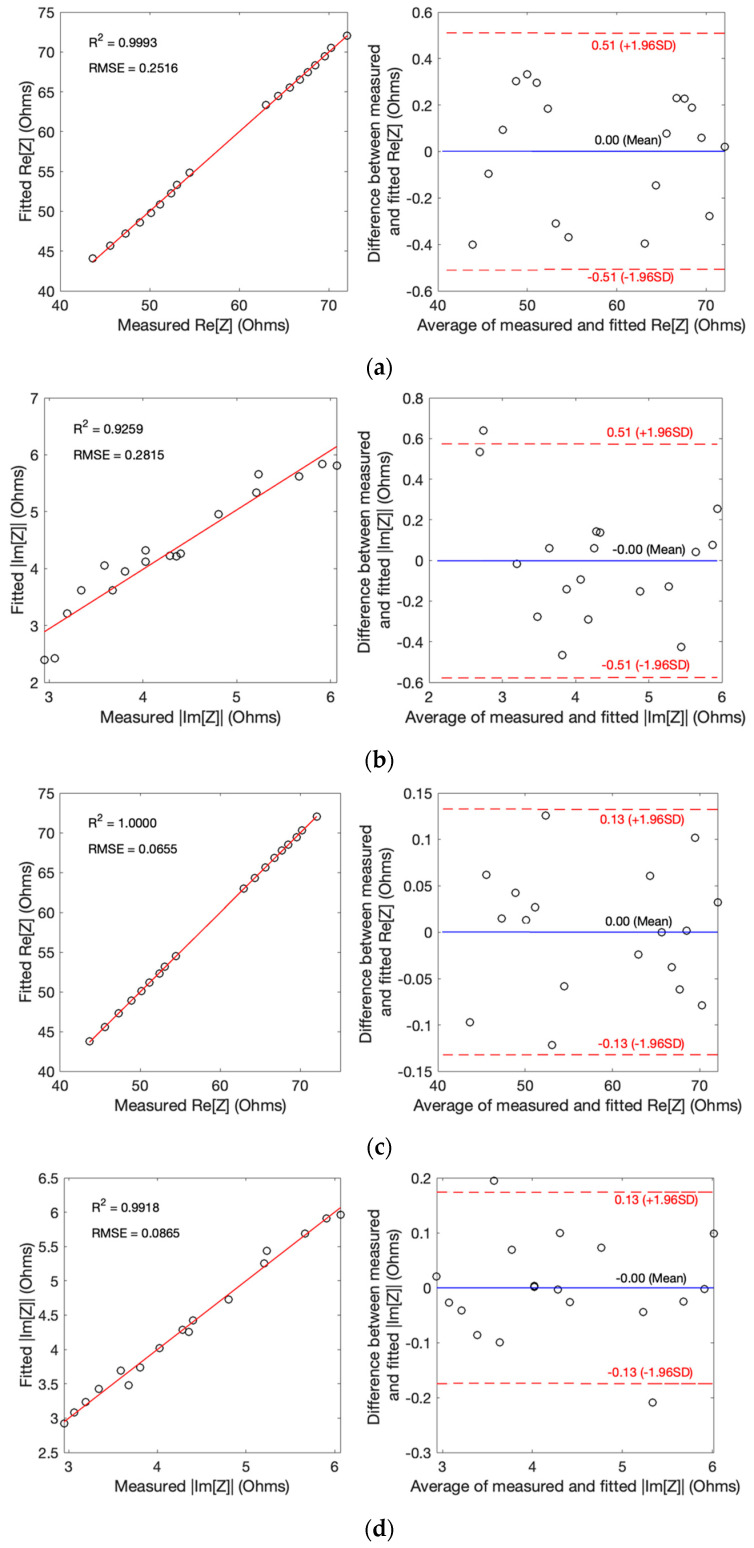
Correlation and Bland–Altman plots for measured impedance: (**a**) real part of impedance using SDCM; (**b**) imaginary part of impedance using SDCM; (**c**) real part of impedance using MDCM; (**d**) imaginary part of impedance using MDCM.

The performance of the fitting process was thoroughly assessed using correlation and Bland–Altman plots, as visually depicted in Figure 7, for both subjects. The collective results underscored a commendable level of accuracy achieved by both models in analyzing the impedance data from the two subjects. Crucially, no significant disparities in fitting quality were discerned between the two subjects.

For SDCM, the analysis revealed an overall *R*^2^ of 0.99 for Re[Z], with a mean difference between the measured raw and fitted values of 0.00 ± 0.26 Ω, signifying a strong fit. Both the RMSE and MAE exhibited reasonable error magnitudes, measuring at 0.25 Ω and 0.0029 Ω, respectively. Similarly, the fitting accuracy for Im[Z] was well established with an *R^2^* of 0.92 and a mean difference of 0.00 ± 0.26 Ω, albeit with slightly higher RMSE and MAE values of 0.28 and 0.0096 Ω, respectively.

In comparison, the MDCM outperformed SDCM, offering even more precise estimations for all measured impedance data from both subjects. The overall *R^2^* were an impressive 1.00 for Re[Z], accompanied by a smaller mean difference between measured raw and fitted values of 0.00 ± 0.06 Ω. RMSE and MAE values were consistent with high accuracy, measuring at 0.06 Ω and 0.0007 Ω, respectively. Furthermore, Im[Z] demonstrated a similar degree of accuracy, characterized by a *R*^2^ of 0.99 and a mean difference of 0.00 ± 0.06 Ω, albeit with slightly higher RMSE and MAE values of 0.08 and 0.0008 Ω, respectively.

## 4. Discussion

### 4.1. Simulation Analysis

In this study, we initially conducted an FEM simulation using ANSYS HFSS to gain a deeper understanding of the E-field distribution within the entire forearm model. As illustrated in Figure 3, the simulation results confirmed a higher E-field concentration around the current-carrying electrodes, consistent with prior research findings [13,28]. Notably, it was found that resistive effects were predominant across the investigated frequency range. An intriguing observation from the simulations was the presence of Cole behavior above 10 kHz. However, an overlap between the dispersion regions was noticed, indicating that the 3D forearm model did not exhibit the typical β-dispersion behavior below 10 kHz, as commonly seen in the prototypical semi-circular Cole plots. This deviation can be attributed to the overlapping α-dispersion and β-dispersion frequency regions, as well as the dominant resistive behavior of blood. This discrepancy was a key factor contributing to the lower fitting performance observed in the simulation study. To enhance the fitting quality, we propose narrowing the fitting frequency range from 10 kHz to 1 MHz.

### 4.2. Pilot Experimentation 

Additionally, we conducted MF-BIA on two healthy subjects to corroborate the findings obtained from the simulation. The Quadra^®^ device is effective at its specified frequencies, which showed a reasonable Cole-type dielectric response in the way of the semi-circular shape of the major portion, while it was limited in its upper-frequency capability. To overcome this limitation and access impedance data spanning from 1 kHz to 1 MHz, we turned to extrapolation methods. This extension of the frequency range allowed us to gain insights into the behavior of biological tissues across a wider spectrum to match the simulated frequency range and enhanced the accuracy and applicability of our impedance analyses.

Consistent β-dispersion behaviors were observed in both subjects. In the comparison of measured Re[Z] between subjects, subject 1 consistently displayed higher impedance values across the measured frequency range, spanning approximately 76 Ω to 61 Ω, while subject 2 exhibited impedance values ranging from 58 Ω to 42 Ω. These disparities in impedance can be ascribed to variances in BMI and forearm circumference, as outlined in Table 1. Subject 1, characterized by a higher BMI, likely possesses a larger volume of radius and ulna bones and a higher proportion of body fat. This resulted in elevated impedance values spanning from 1 kHz to 1 MHz, primarily attributed to the lower electrical conductivity of bone cancellous (0.08 S/m), bone cortical (0.02 S/m) and fat tissue (0.02 S/m). Conversely, subject 2 exhibited a broader impedance change range of 17 Ω, possibly indicating a higher proportion of muscle tissue in the forearm. Muscle tissue displays more significant conductivity variations, ranging from 0.3 S/m to 0.5 S/m between 1 kHz and 1 MHz. However, it is vital to acknowledge that the measured impedance values can be influenced by additional factors, including hydration levels and variations in other tissue compositions, necessitating further investigation to comprehensively understand the multifaceted nature of impedance differences.

Significantly, the magnitudes of the measured impedance spectra were notably smaller compared to the simulation results, a finding consistent with the expected disparities between the 3D forearm model and real human tissues. In light of this, we have consolidated further disparities between the simulation setup and actual measurements in Table 6. While replicating the intricacies of the human forearm precisely in simulation presents challenges, our simplified model has nevertheless yielded valuable insights into the E-field distribution within the tissues. Furthermore, both the simulation and pilot study confirm the Cole-type behaviors exhibited by forearm tissues under BIM.

### 4.3. Electrical Modelling

In this study, a two-stage methodology was adopted to model and analyze the MF-BIA response from the computational simulation and pilot experimentation to estimate the impedance contribution from each tissue domain, giving insights into not only the resistance contribution of each tissue, but also the other Cole dispersion parameters.

Table 7 and Table 8 offer a comprehensive overview of the performance of both models throughout the various investigations conducted in this study. Initially, a broader frequency range spanning from 1 kHz to 1 MHz was simulated. However, as previously discussed, anomalies were observed, particularly a derivative below 10 kHz, which adversely affected the fitting performance of both models. Despite the previous validation of the Quadra^®^ device in prior studies [15,29], we identified abnormal measurements above 179 kHz, prompting the removal of these abnormal data points from the fitting process. To address the limitation imposed by the measured frequency range of the Quadra^®^ device, we undertook the estimation of the complete response, allowing us to gain further insights and a more comprehensive understanding of the dielectric behaviors of the human forearm across the frequency spectrum from 1 kHz to 1 MHz.

Compared with the Im[Z], both models showed better estimation in Re[Z], a phenomenon influenced by various factors. In the context of biological tissues, the resistive component (real part) of impedance normally dominates over the capacitive component (imaginary part) at lower frequencies, associated with the flow of ions or current through tissues. Conversely, the capacitive behavior is related to the accumulation and discharge of charge in cell membranes, which becomes more prominent at higher frequencies. Additionally, Re[Z] is often easier to model accurately since it is relatively straightforward to represent with the Cole model framework. On the other hand, the imaginary part involves capacitance and inductance, which may require more complex models to capture accurately.

The MDCM consistently exhibited superior accuracy across multiple evaluation metrics, including R^2^, EMSE, MAE, mean difference and SD, when compared to the SDCM. These findings underscore the robustness of the MDCM in representing the complex dielectric properties of various tissues, both in the context of simulated and measured impedance values. Every tissue domain experiences a different dispersion phenomenon and hence may not be accurately described using the SDCM. More importantly, the MDCM can describe and estimate the Cole parameters of each tissue, thereby isolating the behavior of a single tissue of interest from the overall MF-BIA.

To rigorously evaluate these models, we conducted statistical comparisons (a paired *t*-test) based on the RMSE of their respective fits to the data. The results of our statistical analysis indicated that there was no significant difference between the SDCM and MDCM (*p* = 0.1055 for Re[Z], 0.0913 for Im[Z]) based on the RMSE measure, suggesting that both Cole models performed comparably in fitting both simulated and measured bioimpedance data, and there was no compelling evidence to favor one over the other in terms of goodness of fit. The overall performance was promising, which can be helpful in physiologically monitoring an organ or a section of the human body through MF-BIA applications, such as electrical impedance tomography (EIT). Furthermore, it can help improve the accuracy of existing methods, like impedance cardiography (ICG) for hemodynamic monitoring by filtering out the impedance contributions from the surrounding tissues to blood-flow-induced impedance variations.

### 4.4. Limitation

This study provides a better insight and understanding in multi-tissue Cole modeling, encompassing simulations and measurements from two subjects. Nevertheless, it is imperative to acknowledge that the outcomes, whether stemming from simulations or experiments, are contingent on specific assumptions, including the assumption of constant tissue properties and simplified geometric representations, while facilitating the study, may introduce inherent limitations, necessitating thoughtful consideration during result interpretation.

It is vital to delineate the disparities between the simulated 3D forearm and the actual anatomical structure. Consequently, several potential limitations merit attention, and future efforts should aim to address them comprehensively. The omission of the skin layer in this study was a deliberate simplification aimed at reducing model complexity. This decision was grounded in the assumption that the use of wet electrodes predominantly mitigates the effects of skin–electrode polarization. Moreover, dynamic factors such as blood flow, which significantly influence the dielectric response of the human forearm, were not considered in this study. The focus was primarily on modeling the distinct compositional responses of muscle, fat, bone and blood-filled artery domains, isolating the blood contribution from the overall measurements.

A promising trajectory for future refinement entails the enhancement of the simulated 3D forearm model geometry. Subsequent research endeavors could enrich this model by incorporating additional tissue components and refining the geometric representation to more faithfully replicate the intricate forearm structures observed in human subjects. Furthermore, expanding the subject pool in future experiments and harnessing advanced imaging technologies, like ultrasound and magnetic resonance imaging (MRI), could prove instrumental in obtaining comprehensive data regarding tissue proportions within subjects’ forearms. This approach holds the potential to yield profound insights into the influences of various tissue types and their proportions on BIA.

More importantly, the mathematical models employed in this study exhibited imperfect fitting for Im[Z]. To address these differences in fitting performance, it is expected to fine-tune the models by employing more sophisticated modeling techniques or to explore alternative models that can better capture the behavior of both Re[Z] and Im[Z], such as the non-linear least-squares fitting technique, which enables the extraction of double-dispersion Cole impedance parameters without relying on direct impedance or phase information [45]. Additionally, fractional calculus has been applied to model biological systems, providing accurate yet concise representations [46,47]. Another notable research effort introduced a novel parametric-in-time approach for electrical impedance spectroscopy, tailored for time-varying impedance systems. This approach was validated through in situ measurements of in vivo myocardial impedance and offers valuable insights into periodic time-varying behaviors [48]. Additionally, improving the accuracy of impedance measurements and reducing noise can also lead to better estimation results for both components of impedance.

## 5. Conclusions

This study has delved into MF-BIA with a multifaceted approach, encompassing computational simulations and real measurements of forearm impedance. We have elucidated the capabilities and limitations of both SDCM and MDCM in characterizing the electrical properties of forearm tissues. Leveraging extrapolation techniques, we extended the frequency range of BIM, facilitating a more comprehensive understanding of tissue behavior. Our investigation also shed light on the influence of factors such as BMI, forearm circumference and tissue composition on impedance data, further underscoring the complexity of bioimpedance phenomena. Notably, the MDCM emerged as a superior modeling approach, offering enhanced precision in estimating impedance parameters. While this investigation marks significant progress, further analysis and refinements remain imperative to fully unlock the potential benefits of the MF-BIA methodology over existing BIA techniques. Ultimately, these efforts hold the promise of standardizing and enhancing the current applications of MF-BIA, paving the way for its broader adoption and impact in diverse fields.

## Figures and Tables

**Table 1:** Subject information.

	Age	Gender	Height	Weight	Body Mass Index (BMI)	Forearm Circumference of Measured Region
Subject 1	32	Male	182 cm	110 kg	33.2	19.3 cm to 26.5 cm
Subject 2	27	Male	188 cm	103 kg	29.1	17.1 cm to 23.8 cm

**Table 2:** Estimated Cole parameters from the fit of simulation data using SDCM.

$R_{0}$	$R_{\infty }$	α	τ
89.50	60.02	0.6923	3.45 × 10^−7^

**Table 3:** Estimated Cole parameters from the fit of simulation data using the MDCM.

$R_{bone}$	$R_{0f}$	$R_{\infty f}$	$\alpha_{f}$	$\tau_{f}$	$R_{0m}$	$R_{\infty m}$	$\alpha_{m}$	$\tau_{m}$	$R_{0b}$	$R_{\infty b}$	$\alpha_{b}$	$\tau_{b}$
49.72	19.72	2.43	0.34	1.48 × 10^−9^	21.44	0.98	0.83	4.08 × 10^−7^	4.04	0.54	0.26	1.05 × 10^−4^

**Table 4:** Estimated Cole parameters from the fit of experimental data using the single-dispersion Cole model.

	$R_{0}$	$R_{\infty }$	α	τ
Subject 1	75.29	56.32	0.54	4.00 × 10^−6^
Subject 2	56.59	35.18	0.64	2.73 × 10^−6^

**Table 5:** Estimated Cole parameters from the fit of experimental data using the multi-dispersion Cole model.

	$R_{bone}$	$R_{0f}$	$R_{\infty f}$	$\alpha_{f}$	$\tau_{f}$	$R_{0m}$	$R_{\infty m}$	$\alpha_{m}$	$\tau_{m}$	$R_{0b}$	$R_{\infty b}$	$\alpha_{b}$	$\tau_{b}$
Subject 1	57.98	25.51	16.09	0.65	7.29 × 10^−5^	24.50	15.82	0.67	6.91 × 10^−5^	3.54	0.62	0.62	1.63 × 10^−4^
Subject 2	38.83	25.59	12.30	0.72	2.32 × 10^−4^	26.80	14.59	0.88	3.91 × 10^−5^	2.86	0.53	0.66	6.28 × 10^−4^

**Table 6:** Comparison between simulation and forearm measurements.

	Computational Simulation	Pilot Study
**Skin–electrode contact interface**	No contribution of the electrode interface (polarization) was considered in the overall measurements.	The measurement device and the gel electrodes provided an appropriate compensation for the skin–electrode interface.
**Size of electrodes**	The electrodes had the same cross-sectional contact dimensions to the dry electrode (diameter = 8 mm).	The gel electrolyte between Ag/AgCl electrode and the skin provided a larger contact area.
**Tissues**	Only four tissues were constructed (i.e., fat, muscle, bone and blood). All modelled tissue domains were relatively uniform with bulk dielectric properties.	The anatomy of the forearm (dimension and proportion) was different between each subject. Other tissues contributed to the overall BIM, such as tender, vein, nerve, etc.

**Table 7:** Summary of statistical analysis of proposed electrical models for the real part of impedance.

		Correlation Analysis		Bland–Altman Analysis		Mean Absolute Error [Ω]
		R^2^	RMSE [Ω]	Mean Difference [Ω]	SD [Ω]	
**Simulation**	SDCM	0.98	0.85	−0.06	0.88	0.0588
	MDCM	0.99	0.39	0.00	0.40	0.0006
**Pilot Study**	SDCM	0.99	0.25	0.00	0.26	0.0029
	MDCM	1.00	0.06	0.00	0.06	0.0007
Mean of all SDCM		0.98	0.55	−0.03	0.57	0.0308
Mean of all MDCM		0.99	0.22	0.00	0.23	0.0007

**Table 8:** Summary of statistical analysis of proposed electrical models for the imaginary part of impedance.

		Correlation Analysis		Bland–Altman Analysis		Mean Absolute Error [Ω]
		R^2^	RMSE [Ω]	Mean Difference [Ω]	SD [Ω]	
**Simulation**	SDCM	0.54	1.75	1.13	0.88	1.1303
	MDCM	0.79	1.04	0.56	0.40	0.5570
**Pilot Study**	SDCM	0.92	0.28	0.00	0.26	0.0096
	MDCM	0.99	0.08	0.00	0.06	0.0008
Mean of all SDCM		0.73	1.01	0.56	0.57	0.5699
Mean of all MDCM		0.89	0.56	0.28	0.23	0.2789

## Data Availability

Not applicable.
